# Fit for purpose: evaluation and evolution of agricultural bioinoculants across production landscapes

**DOI:** 10.1093/ismeco/ycag190

**Published:** 2026-07-10

**Authors:** Lauren M Hemara, Rebecca T Doyle, George C diCenzo, Matthew G Bakker, Kailey R Hopkins, Jangwoo Lee, Ivan J Oresnik, Terrence H Bell

**Affiliations:** Department of Physical and Environmental Sciences, University of Toronto Scarborough, Toronto, ON M1C 1A4, Canada; Department of Biology, McMaster University, Hamilton, ON L8S 4K1, Canada; Department of Biology, Queen’s University, Kingston, ON K7L 3N6, Canada; Department of Microbiology, University of Manitoba, Winnipeg, MB R3T 2M5, Canada; Department of Microbiology, University of Manitoba, Winnipeg, MB R3T 2M5, Canada; Department of Physical and Environmental Sciences, University of Toronto Scarborough, Toronto, ON M1C 1A4, Canada; Department of Biology, McMaster University, Hamilton, ON L8S 4K1, Canada; Department of Microbiology, University of Manitoba, Winnipeg, MB R3T 2M5, Canada; Department of Physical and Environmental Sciences, University of Toronto Scarborough, Toronto, ON M1C 1A4, Canada

**Keywords:** plant growth-promoting microorganisms, microbial inoculants, trade-offs

## Abstract

Microbial inoculants are currently advancing sustainable agriculture by reducing reliance on chemical fertilizers and pesticides, and enhancing soil and plant health in ways that traditional chemical inputs cannot. However, despite over a century of research and development, their in-field establishment and performance remain unreliable, limiting their widespread adoption and impact. Typically, the selection of candidate inoculants centers around performance-based traits (e.g. nitrogen fixation) assessed *in vitro*; yet, viable and impactful inoculants must express a wide range of traits that promote their fitness across heterogeneous landscapes. Successful inoculants must do well across the industrial production pipeline, which requires rapid growth in nutrient-rich liquid media. They must also tolerate long-term storage and subsequently perform consistently across diverse field conditions. These traits are essential for scalability, economic viability, and impact. To date, commercially available inoculants are mostly based on microbes exhibiting rapid growth under common laboratory conditions, traits which may or may not correlate with in-field delivery of beneficial functions. In this review, we propose a multi-faceted evaluation approach for inoculant performance, considering both biotic and abiotic aspects of inoculant success. We then use this framework to evaluate how adaptive laboratory evolution could enhance inoculant performance at key industrial pipeline steps. This approach is essential to widening the range of taxa that are considered for commercialization, while identifying and potentially mitigating the pitfalls of growing, storing, and applying microbes through traditional industrial production pipelines.

## Introduction

Many growers rely on chemical nutrient and pesticide inputs, because they are easy to apply and (generally) predictable in their beneficial effects on crop yield. However, the negative consequences of such inputs are well known, including excess nutrient loading in nearby waterways and risks to biodiversity and human health [1]. For more than a century, a promising alternative to chemical inputs has been explored: microbial inoculants. Microbial strains are isolated from the environment, screened, and then grown to high concentrations in batch culture, before being applied to seeds, plants, or soils. In laboratory settings, various strains have been repeatedly shown to enhance functions that are beneficial to soils or plants, such as promoting plant growth, increasing water uptake, controlling pests and pathogens, remediating soil contaminants, or generally improving soil structure and health [2]. In medicine, inoculated microbes have led to reliable improvements in human health status, including the use of fecal transplants to successfully treat recurrent *Clostridioides difficile* infection [3]. However, in agriculture, beneficial laboratory results often do not translate to real-world yield increases and agronomic impact [4, 5]. The unpredictable establishment and function of microbial inoculants in the field is a significant barrier to widespread adoption [6, 7]. This challenge is exemplified by the highly variable establishment outcomes of a commercial phosphate-solubilizing *Priestia megaterium* inoculant, which either thrived, persisted, or rapidly declined when introduced into soils collected from within a relatively small geographic area (~10-mile radius) [8].

Current inoculant development efforts have largely focused on identifying microbes with desired plant growth-promoting (PGP) traits *in vitro*. For instance, if the goal is to enhance N_2_ fixation, successful candidate inoculants are those that perform this function best in screening assays. However, most PGP traits are not especially rare; one could easily find above-average N_2_ fixers in their backyard or at a city park. Perhaps more important, and certainly more complicated, is to ensure that a candidate inoculant both possesses the desired trait(s) and maintains fitness across variable environments. This includes the entire industrial production pipeline—from growth in batch culture to field application—as well as the diverse and fluctuating environmental conditions encountered through space and time post-application, even within a single farm. For instance, a microbe cannot be advanced from initial isolation to in-field application, without passing through batch culture in between. For agricultural inoculants to become true alternatives to chemical additives, we must prioritize their compatibility with all of the conditions they will be exposed to throughout the production pipeline, explore potential trait trade-offs across environments, and better align *in vitro* evaluation with *in situ* success [6].

In this action-focused review, we outline strategies for producing and evaluating inoculants that can withstand complex agroecosystem and production environments, leveraging advances in microbial genomics and adaptive laboratory evolution (ALE). Despite the increasing use of microbial inoculants, current regulations and standards rarely require inoculant products to demonstrate quality control or environmental compatibility—that is, growth and function under field conditions—limiting their reliability in diverse field settings. Because growers cannot afford to adopt unreliable products, a multi-faceted evaluation of inoculant survival and performance is essential to encourage broader adoption of technologies based on live microbial inoculants.

## A framework for evaluating the breadth and resilience of microbial inoculants

### Evaluating both the abiotic and biotic aspects of inoculant success

**Figure 1:**
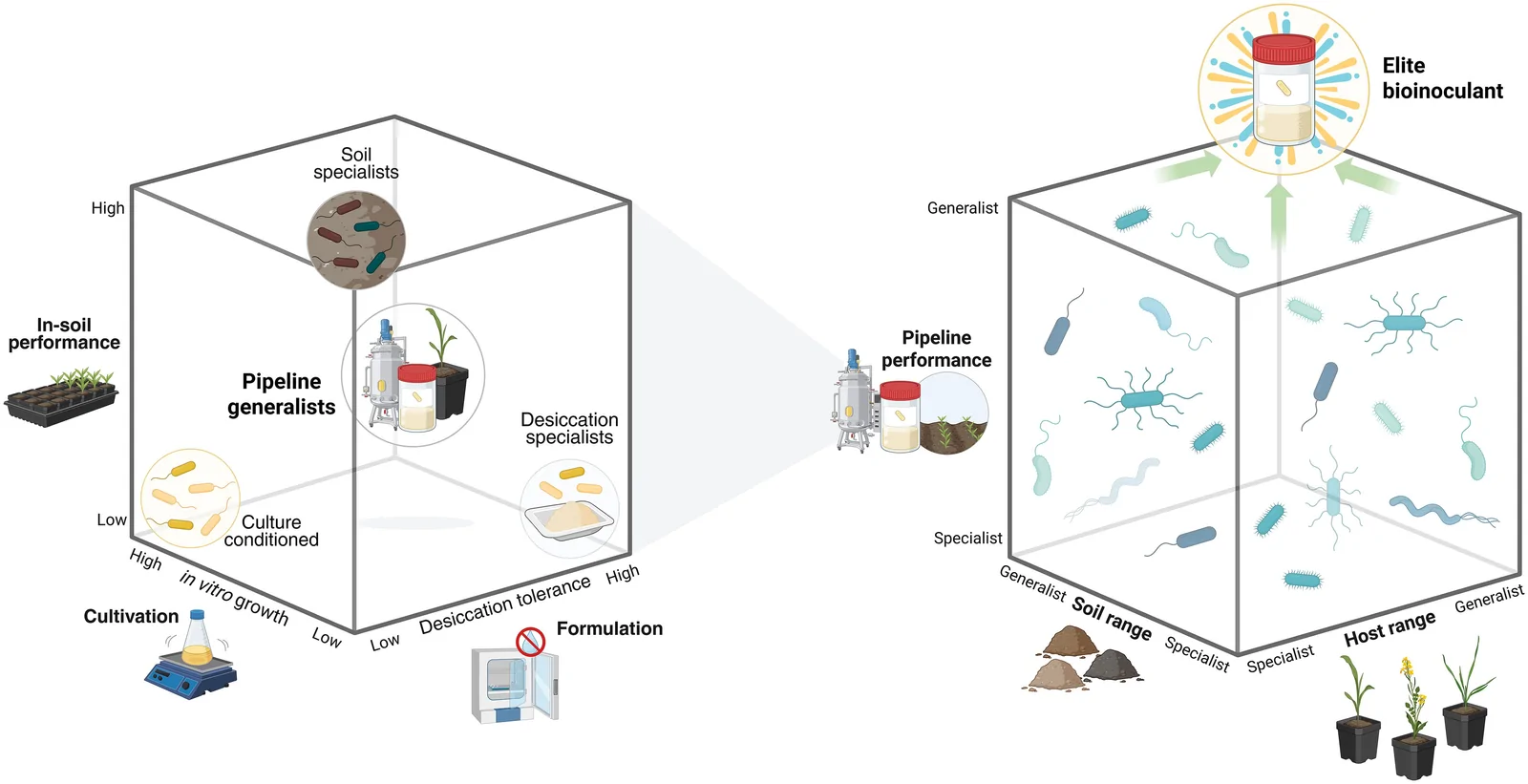
The broad environments found across industrial and agricultural production landscapes and relevant traits for elite performance. Left: potential inoculants may be specialists or generalists for any given production landscape, as depicted on the axes. Pipeline generalists are expected to exhibit high *in vitro* growth, desiccation tolerance, and in-soil growth, whereas specialists may have superior performance in only one of these environments. Right: high performance across the industrial pipeline itself is just one of the traits of interest an elite bioinoculant may require. Additionally, elite performance may require inoculants to be a soil type or host range generalist to establish reliably in diverse field conditions.

Inoculant development approaches must bridge the current disconnect between *in vitro* evaluation and *in situ* efficacy, which requires evaluation of diverse survival traits. Inoculant evaluation that screens for both industrially relevant traits and environmental compatibility would drive commercial viability. Specifically, performance must be considered across key production landscapes, including in-culture growth during batch cultivation, long-term viability during the formulation process, and in-soil success across diverse recipient field conditions. To identify microbial candidates for further evaluation and adaptation, preliminary screening of a microbial library should focus on characterizing performance across these landscapes to identify production pipeline generalists (with high fitness across multiple environments) or specialists (with superior fitness in specific environments) (Fig. 1). Existing studies have examined these microbial traits relevant to production in isolation, including growth during cultivation [9–18], sporulation efficiency [15, 16, 19–22], storage and formulation tolerance [23–32], in-soil growth and survival [8, 33–39], and PGP efficacy following application [2, 40–42].

However, current literature lacks explicit connection and comparison of these traits across production landscapes. Breaking down these silos is also increasingly feasible, as high-throughput platforms now exist for many of these assays. For instance, automated microplate assays can quantify bacterial growth in-culture and survival rates under various abiotic conditions, including prolonged storage. Furthermore, commercial products like the EcoPlate™ and Odin™ instrumentation (Biolog®), or the MicroResp™ soil respiration system, allow for high-throughput evaluation of resource utilization and metabolism *in vitro* and in-soil [33, 43, 44]. Moreover, evaluating microbial traits sequentially across production stages is critical, as high performance in one production phase does not guarantee, and could in fact compromise, success in the next. For example, a microbe that grows well in culture and in soil may still fail as an inoculant if it cannot tolerate nutrient starvation or survive shelf storage, preventing it from ever reaching the field. Additionally, microbes can retain cellular “memory” of their previous environment, where some of the population still expresses transcriptional programs in response to a previous stimulus or environment [45]. This could mean that the conditions microbes experience throughout the production pipeline may influence their downstream performance in the field.

Another hurdle in the production of reliable inoculants is the lack of an explicit efficacy baseline: a minimum threshold of survival and function required across production environments to ensure predictable in-field benefits. This challenge is exemplified by the quality control issues that have been widely identified in commercial arbuscular mycorrhizal fungi (AMF) products [41, 46]. A recent meta-analysis revealed that 84% of these products fail to colonize plant roots under controlled conditions, suggesting that farmers may be wasting $876 million USD annually on ineffective products [46]. These systemic failures underscore a broader issue within the industry that likely extend to other fungal and bacterial inoculants. While quality management criteria and assays have been proposed for AMF products [47], bacterial inoculants would similarly benefit from a robust efficacy baseline—particularly one that evaluates and differentiates between microbial survival and measurable delivery of the target beneficial function(s). The “core competencies” required for this baseline depends (in part) on the microbe’s lifestyle. For example, while free-living phosphate solubilizers or nitrogen fixers need to persist in both liquid culture and soil environments [48], plant endophytes may bypass the need for in-soil establishment, provided they can successfully colonize the plant root upon inoculation [41, 46]. Regardless of their lifestyle, inoculants must navigate the physicochemical and biotic diversity of real-world soils, withstanding the varied combinations of soil properties, resident microbiomes, and target crops (often under additional abiotic stresses like extreme temperatures or drought). Soil texture, pH, nutrient availability, minerals, metals, salinity, moisture, and temperature can all drive the assembly of soil microbiomes, and thus can impact both the establishment and functioning of inoculants [2, 42, 49–53]. Ecological factors can also influence inoculant establishment, including compatibility or competition with resident microbiomes, the open niche space available in a given soil, and the timing of introduction—a factor which often dictates both community composition and niche availability [54–56]. Diverse field conditions must therefore be incorporated into the evaluation process to better define the environmental range of candidate inoculants [57].

We propose that once candidate inoculants have been identified based on industrially relevant traits, these candidates then be assessed for compatibility with physiochemically diverse soils and diverse resident microbiomes to determine whether they have the potential to thrive across variable conditions present within a single farm or across a geographic region of interest. Screening candidate inoculants for compatibility with diverse soil microbiomes will further improve our understanding of whether inoculants can reliably engraft into all (or only some) resident microbiomes and identify key microbe–microbe interactions that impact the probability of establishment. Similarly, inoculants can be assessed in terms of their compatibility across diverse target crops, to determine the full scope of potential application. All of these fitness and environmental breadth evaluations can be integrated with genomic data to identify genetic markers associated with key traits [58, 59], allowing us to build predictive models that match inoculants to compatible environments. Such an approach could lead to more informed inoculation strategies by tailoring product use to the specific crop, soil, and climate conditions of a target site. However, a balance must be struck between a “one for all” solution and a hyper-localized approach (e.g. bespoke inoculants for any given field); realistically, a limited set of products tailored for a limited set of conditions would be required for farmers and consultants to implement an informed inoculation approach [57].

### Evaluating trade-offs across production landscapes

Ultimately, while inoculants must be suitably adapted to the industrial production pipeline, potential beneficial functions must be realized in-field for an inoculant to be effective [60]. The same features that make microbes great models for experimental evolution—including rapid growth in liquid culture—can make them challenging to manage through an extended production pipeline. If microbes are growing, they are evolving, which can lead to unintended changes in function or fitness at different stages. For example, if traits required for in-soil survival, dispersal, and plant growth promotion are selected against during industrial production, this endangers the commercial viability of a candidate inoculant.

Each production stage also represents a unique environment that imposes new selection pressures on an inoculant (Fig. 1) and dictates which are best adapted. For instance, commercial inoculants must be able to grow in high-nutrient liquid media during batch cultivation *and* under nutrient- and water-limited soil conditions in the field [6, 29]. For the phosphorous-solubilizing species *P. megaterium*, batch culture has been shown to reduce fitness in certain soils downstream [61], highlighting just one of the intrinsic conflicts of industrial inoculant production [6]. The formulation process presents another fundamentally different environment, and introduces further abiotic stressors, where inoculants may be formulated as dry powder, granules, or liquid products [62]. In dry formulations, microbes can face extreme desiccation stress [31, 63]. While some bacteria (e.g. *Bacillus* spp.) can produce stress-tolerant spores that withstand desiccation stress, other microbes rely on alternative strategies (such as the accumulation of osmoprotectants) to stabilize desiccated cells during physiological stress [64–67], or otherwise perish*.* Moreover, the desiccation tolerance required for dry formulation has the potential to conflict with traits required for batch cultivation or growth in water-saturated soils [31, 32, 68]. For microbes that cannot tolerate desiccation, alternatives like liquid formulation may not entirely circumvent the stresses microbes experience during storage. Even in liquid products, microbes must persist long-term in spent media and tolerate storage under fluctuating temperatures between manufacture, retail, and on-farm storage. We have personally seen commercial inoculants stored by farmers under all sorts of conditions, while industry colleagues have referenced the need for “tin shed tests”; essentially, confirming that inoculants are likely to remain viable even when thrown in a tin shed in mid-summer. Few studies to date have captured these trade-offs in the context of industrial production or outside of a single isolate. Fitness evaluations should be designed to identify potential traits that are in conflict across production stages that may impede inoculant survival and efficacy. If we can identify said trade-offs, we may then be able to alleviate them by modifying production processes [6], or altogether circumvent them through trait-guided selection of candidate inoculants.

## Microbial “breeding”: which traits are good targets?

### Microbial “breeding” through adaptive laboratory evolution

Following fitness evaluations across production landscapes, potential inoculants could be further improved through artificial selection to “breed” microbes for desirable traits. In plants, selective breeding has developed wild teosinte into elite maize cultivars with desirable traits. Similarly, we propose that ALE may be able to develop elite microbial inoculants from wild soil isolates—so-called “microbial breeding”. ALE leverages iterative selection to improve performance [69], and relies on the emergence of natural genetic variation either through de novo mutations or selection on existing variation within a strain population. This approach mimics conventional breeding, and contrasts with directed genetic modification and gene-editing technologies that often face greater challenges related to public acceptance and regulatory approvals in many countries.

### Precedent for adaptive laboratory evolution to improve inoculant performance

Microbial ALE has long been used to observe the outcomes of rapid evolution in controlled settings and develop strains with improved traits [69–72]. In laboratory environments, microbial adaptation has been frequently observed in batch culture conditions, resulting in (inadvertent) laboratory “domestication” [73–76]. Most deliberate trait improvement experiments have focused on microbial “bio-factories”, improving biological functions to meet manufacturing requirements and increasing microbial tolerance of cultivation conditions. In this context, both single isolates and consortia have been evolved toward diverse outcomes, including enhanced sugar production and fermentation, nitrogen assimilation, and stressor tolerance (e.g. inhibitory compounds, alcohols, temperature, and salinity) [77–86]. Given this precedent, candidate inoculants may similarly be amenable to selection regimes to create elite strains. However, unlike microbes used for biomanufacturing or industrial-scale fermentation—which are typically only required to adapt to a single environment—inoculants must tolerate multiple conditions. Initial batch culturing of inoculants may resemble biomanufacturing, as commercial microbial products must be grown in production-scale reactors to reach sufficient biomass [87]. However, adaptation to this cultivation stage must not conflict with traits required for the downstream storage tolerance and, ultimately, compatibility with and function within the recipient field.

Recent research has further highlighted the potential of ALE to alter traits relevant to environmental range and host compatibility, particularly in plant–soil systems. For instance, microbial symbionts can rapidly adapt to specific hosts following repeated ALE, leading to improved root colonization or enhanced mutualistic interactions [88–95]. In particular, increased biofilm formation (with a motility trade-off) has emerged as a key trait for improved root colonization by *Bacillus* spp. across several ALE studies [89, 92, 94–96]. Soil-evolved isolates also demonstrate increased soil fitness compared to the ancestor [97–99]. Success is not always guaranteed, however. Microbial responsiveness to ALE can be as unpredictable as their performance in the field, varying widely depending on environmental context and stochasticity of mutations. Bacteria evolved in sterile, competitor-free soils may or may not rapidly shift their in-soil range, depending on the species [99]. We see the same diverse outcomes for symbiont adaptation—rhizobium evolution on *Lotus japonicus*, for example, only enhanced benefits for certain host genotypes [100]. Another disadvantage—and potential risk—of selecting for improved host colonization is that it may (inadvertently) do more harm than good. As host colonization is a trait shared by both symbionts and would-be pathogens, it may take relatively little innovation for an evolved strain to transition from a plant beneficial lifestyle to a detrimental one [101].

### How phylogenetically conserved are target traits?

Horizontal gene transfer within microbiomes is another driver of evolutionary innovation, allowing bacteria to rapidly adapt to new environmental niches [102–104]. Some microbial traits (e.g. carbon substrate utilization) are highly phylogenetically dispersed across major phyla [105]. Yet, despite widespread horizontal gene transfer within microbiomes, other microbial traits—such as oxygenic photosynthesis, methanogenesis, and sulfate reduction—appear to be phylogenetically and hierarchically conserved, potentially due to biochemical and genetic complexity and interdependency [105, 106]. Understanding which traits have strong or weak phylogenetic conservation and the degree to which traits are responsive to ALE will underpin our ability to select for specific traits. By attempting to breed different traits across diverse microbial backgrounds, we can determine whether a trait of interest is broadly modifiable across taxa, restricted to a limited subset of microbes, or even if the trait is modifiable at all. Differing responsiveness to selection, or speeds of trait evolution, can have different utilities for inoculant development. Rapidly evolving traits may be modified with little effort but could be unstable in the field, such that in-field evolution results in a loss of trait improvements. This phenomenon has been observed in *E. coli*, where populations are able to rapidly adapt to prolonged nutrient exhaustion; however, these adaptations are highly transient and are rapidly purged from the population when fresh resources are provided [107]. Conversely, traits that are slow to change may be more difficult to modify initially, but their robustness may also offer greater stability of desired traits once refined. Assessing how readily a trait (or, more generally, a strain) can adapt under specific environmental conditions may help to improve inoculant reliability by identifying target traits (and strains) that can be easily enhanced. Suppose desired traits are environmentally sensitive (i.e. plastic phenotypes). In that case, we can improve inoculant reliability by adjusting production practices to reduce certain pressures [6] or by selecting variants with genetic adaptations outside of mutational hotspots that may be prone to reversion [108]. Such hotspots may be recognized by signature motifs, such as simple sequence repeats which mediate hypermutation, or through the observation of highly repeatable mutation events [109, 110].

### Potential passaging approaches for inoculant improvement

**Figure 2:**
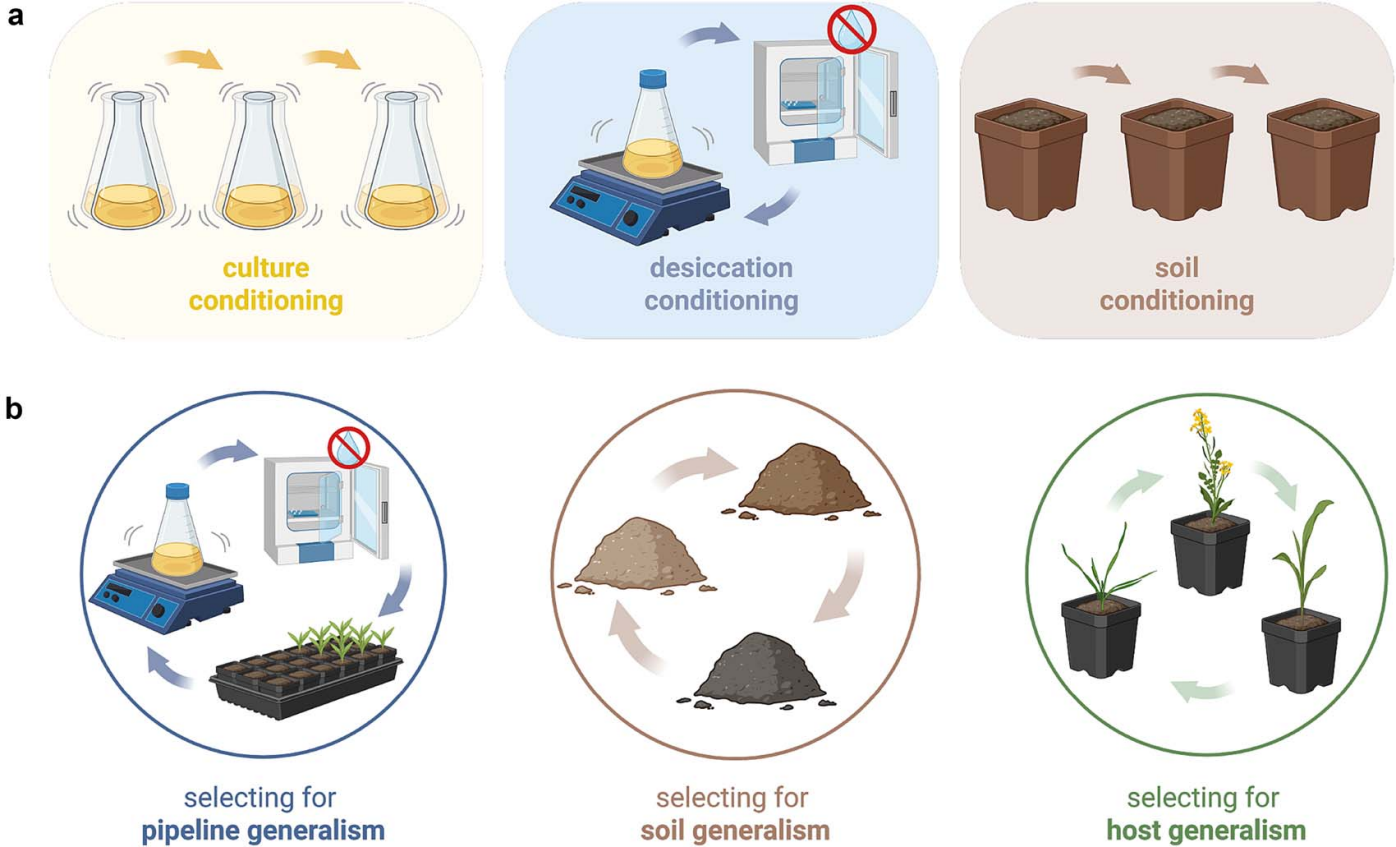
Improving inoculant performance through selective “breeding” across key production landscapes. Conditioning and passaging across the breadth of key environments in these landscapes may allow us to improve inoculant performance by honing relevant traits and broadly selecting for pipeline generalism. (a) Basic conditioning regimes involve repeatedly passaging isolates through a constant environment, including liquid media, desiccation, or soil, allowing for enrichment of naturally-occurring adaptations improving fitness in the environment. (b) Cyclical passaging through the key environments of the industrial pipeline, across physicochemically diverse soils, and through the rhizospheres of target crops may widen environmental breadth.

If screening can identify production pipeline generalists and specialists, ALE offers a mechanism to “breed” elite traits into promising but underperforming candidates. By repeatedly passaging a pure microbial population through one or more environments, lineages with natural mutations improving fitness in the environment(s) are selected and increase in abundance, allowing them to be isolated for downstream work. Through this process, researchers could pick a focal ancestral isolate—which may be a generalist or specialist along a specific environmental axis—and refine or derive a broad range of traits required for inoculant success. For instance, this process of selective “breeding” could increase the environmental breadth of inoculants following repeated passaging through key environments (Fig. 2). We hypothesize that if isolates perform poorly at a specific production stage, targeted passaging in a controlled environment that mimics that stage—such as liquid culture, desiccation with intermittent rehydration, or a single soil source—may help adapt them for improved performance (Fig. 2a). For example, an ALE regime for isolates that initially demonstrate poor desiccation tolerance could involve repeatedly exposing this isolate to desiccation conditions with increasing “dry” periods before rehydration. Furthermore, repeated cycling across diverse conditions could select for generalists that can tolerate environmental variation (Fig. 2b). This strategy can be applied whether starting with existing generalists, shifting specialists toward a broader environmental range, or attempting to improve poor-performing isolates. Repeatedly cycling inoculants through the industrial production pipeline, for example, may select for variants that can perform adequately across all stages (Fig. 2a). Similarly, we may be able to use this approach to expand niche breadth in the field, selecting for inoculants that can tolerate diverse soils or associate with diverse crops (Fig. 2a). Collectively, selecting for generalism or widened niche breadth across these environmental axes may bring us a step closer to an elite, reliable inoculant that can survive and consistently deliver PGP benefits across diverse recipient environments.

### Limitations and potential negative impacts of adaptive laboratory evolution

In line with the anticipated trade-offs experienced by wild-type isolates across production stages, environment-specific conditioning may also result in adaptive trade-offs with other essential traits and functions. Laboratory domestication often results in unintended genetic and phenotypic divergence, due to the repeated cycles of cultivation and cryopreservation microbes undergo during long-term use and storage. In *B. subtilis*, laboratory domestication is associated with the loss of biofilm production [111], swarming motility [112], and sporulation [113]—all traits that may be important for in-soil dispersal and survival. We also see (sometimes extensive) divergence in lab-domesticated cultures following prolonged storage. One such example is *Salmonella enterica* serovar Typhimurium, where decades of storage has resulted in changes in genome structure, genome size, gene order, nitrogen and carbon metabolism, motility, competitive fitness, and colony size [114, 115]. Similar changes have been observed following long-term storage or in-culture passaging of *Sinorhizobium meliloti*, *S. enterica*, *Methylobacterium extorquens*, *E. coli*, and *Lactococcus lactis* [74, 75, 116–120]. Conversely, biofilm production is a trait often improved during rhizosphere conditioning; however, adaptation toward biofilm production can impair in-culture growth and survival [121]. This highlights the potential conflicts that must be balanced in experimentally adapting a strain to a single production stage (e.g. industrial cultivation or in-field conditions). To ensure microbial breeding tools are used effectively, we must carefully evaluate these trade-offs to identify where conditioning may improve inoculant performance and where it may have unexpected consequences. Whether it is possible to stack these desirable traits in a single background, or whether trade-offs prevent trait stacking, will be an important discovery for microbial breeding efforts across a myriad of potential industrial applications.

To realize the potential of ALE for inoculant improvement, there are a number of outstanding questions that need to be addressed, including:

- How do the timelines required for ALE align with the development timelines and economic constraints of commercial inoculant production pipelines? ALE may require hundreds or thousands of microbial generations to achieve significant phenotypic shifts, if such shifts are possible at all. Moreover, this is likely to differ by orders of magnitude depending on the focal taxon. In an expanding commercial landscape, time to market may be constrained by the timeline required to realize strain improvement through ALE.
- For which traits can ALE deliver commercially meaningful improvements within realistic timeframes? Currently, trait responsiveness to ALE is poorly documented outside of yield in microbial biofactories. Failed ALE attempts are also likely to be seldom reported.
- To what extent are traits evolved under laboratory conditions stable and beneficial after scale-up, formulation, storage, and field application? A significant risk of ALE is that trait improvement may only be temporary and could quickly revert under selective pressure or genetic drift outside of controlled settings.
- Could laboratory-evolved traits compromise microbial competitiveness or persistence in complex soil environments? Specialization to specific, controlled conditions may result in trade-offs; we have seen this documented extensively in other contexts (including plant adaptation [89, 95] and laboratory domestication [111–113]). If these trade-offs reduce microbial competitiveness, any fitness gains through trait improvement may be outweighed by an inability to survive in the recipient abiotic conditions or to successfully engraft into the resident microbiome.

## Ingredients matter: optimizing the sourcing of microbial inoculants

**Figure 3:**
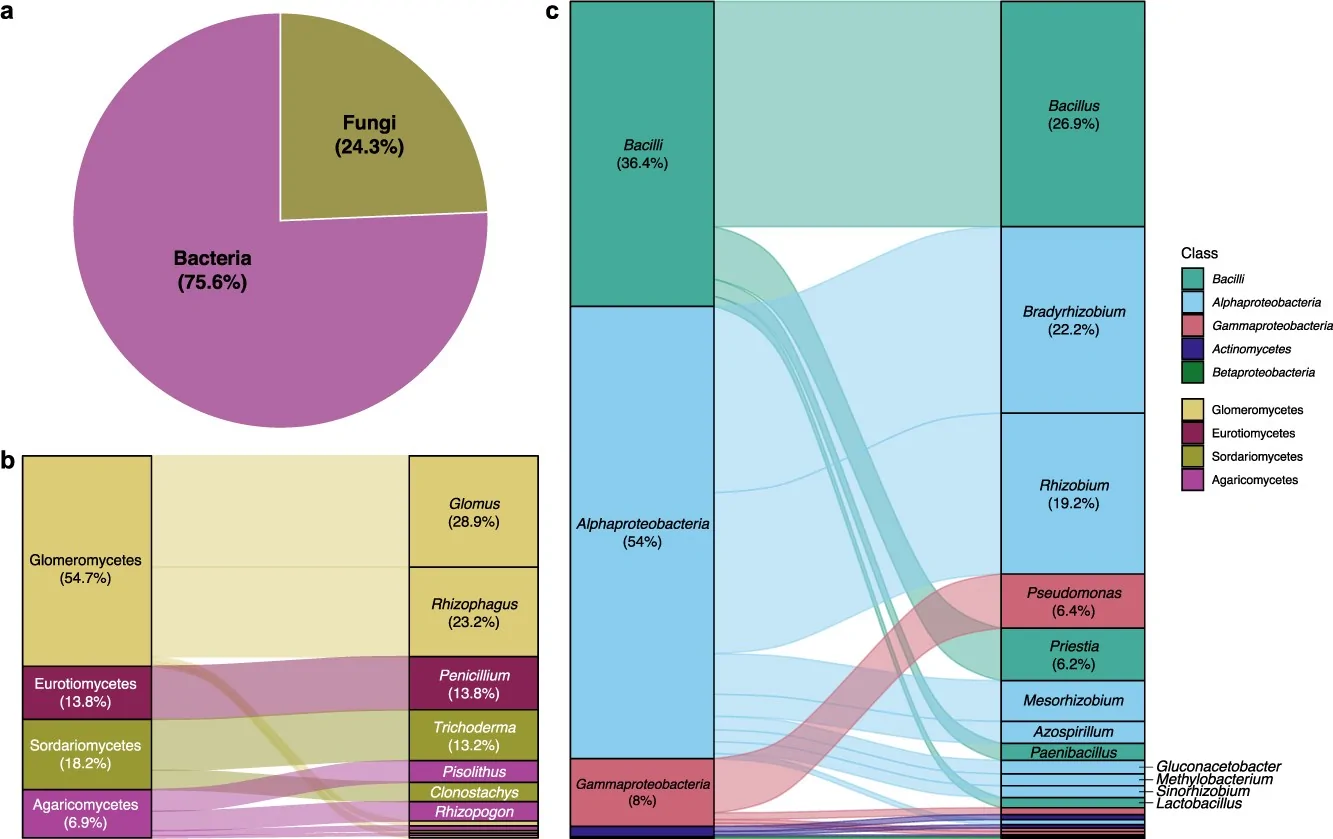
Bacterial and fungal fertilizer products registered with the CFIA. Data was retrieved in October 2025 from the Registered Fertilizer Products List. (a) Percentage of registered products that contain bacteria and/or fungi. (b) Proportion of fungal genera present in registered fertilizer products. (c) Proportion of bacterial genera present in registered fertilizer products.

If key traits cannot be selectively bred, inoculant development may remain reliant on the initial niche breadth of an ancestral isolate and the traits available in the microbial source pool [6]. A relatively small number of soil-dwelling genera currently dominate the microbial inoculant market [122, 123]. Numerous reviews [2, 48, 122, 124] have underscored the global dominance of just a handful of genera in commercial inoculants across root-associated fungi (e.g. *Rhizophagus*, *Glomus*, *Penicillium*, *Trichoderma*), rhizobia (e.g. *Rhizobium*, *Bradyrhizobium*, *Sinorhizobium*), and free-living bacteria (e.g. *Pseudomonas*, *Bacillus*, *Azospirillum*, *Arthrobacter*, *Streptomyce*s). Here, we present a case study from the Canadian biofertilizer market—which has significant overlap with the US market, but with the added benefit of a publicly-available registration database. Just over 20% of the 2100+ fertilizers registered with the Canadian Food Inspection Agency (CFIA) are bacterial or fungal products (Fig. 3a). Of these, 47% of products contain rhizobia, including species from the genera *Bradyrhizobium*, *Rhizobium*, and *Mesorhizobium* (Fig. 3). Beyond root-nodulating rhizobia, *Bacillus* is the most common bacterial genera, present in 26.9% of bacterial fertilizers (Fig. 3c). *P. megaterium* (previously classified within the genus *Bacillus*) and *Pseudomonas* spp. account for another ~6% each (Fig. 3c). In particular, free-living *Pseudomonas* and *Bacillus* are commonly isolated rhizobacteria, with many species considered to be ubiquitous environmental generalists [89, 125]. These species are also overrepresented in commercially available (non-rhizobia) biofertilizers on the Canadian market (Table 1). Taken together, this narrow commercial focus underscores how limited current bioprospecting efforts may be. There may be unexplored PGP and ecological potential among more diverse and often overlooked soil taxa.

**Table 1:** Select biofertilizers commercially available in Canada.

Product	Company	Formulation	Species
LALRISE VITA	Lallemand	Dry powder	*B. velezensis*
Éra Boost Pro	Ulysse Biotech	Liquid	*Bacillus licheniformis, B. velezensis, Bacillus subtilis, Priestia megaterium*
Earth Alive Soil Activator	Earth Alive	Dry powder	*B. subtilis, Bacillus amyloliquefaciens, Pseudomonas monteilii*
AgriFlora PRO	Koppert	Liquid	*B. subtilis, B. amyloliquefaciens*
MIICROBIAL MASS PRO	MIIMHORT	Liquid	*B. velezensis, B. licheniformis, Priestia megaterium*
AGTIV STIMULATE L	Premier Tech	Liquid	*Bacillus inaquosorum*
ACCelerate	Ceragen	Liquid	*Pseudomonas fluorescens*
AL-Bio7	A&L Biologicals	Liquid	*Pseudomonas azotoformans*
PhosN Seed Coating	Biolevel	Dry powder	*Pseudomonas putida, B. subtilis, B. licheniformis, Bacillus pumilus, B. amyloliquefaciens, Priestia megaterium*, *Paenibacillus polymyxa*
PhosBac 45	Andermatt	Liquid	*B. amyloliquefaciens*
RhizoVital 42	Andermatt	Liquid	*B. velezensis*
C5 TundraBac	Andermatt	Liquid	*Bacillus atrophaeus*
PrimAgro P	Agroliquid	Liquid	*B. subtilis, Bacillus methylotrophicus*
PrimAgro N	Agroliquid	Liquid	*B. subtilis*
PrimAgro C-Tech	Agroliquid	Liquid	*B. subtilis, B. amyloliquefaciens*
XiteBio Vegi+	XiteBio	Liquid	*Bacillus firmus*
PGP2R	BioNorth Solutions	Dry powder	*P. putida, Priestia megaterium*
Utrisha N	Corteva	Dry powder	*Methylobacterium symbioticium*
M-BOS	NutriAg	Liquid	*Methylobacterium organophilum*
Tarantula	Advanced Nutrients	Liquid	*Arthrobacter globiformis, Bacillus coagulans, B. licheniformis, Bacillus mucilaginous, B. pumilus, B. subtilis, P. polymyxa*
Voodoo Juice	Advanced Nutrients	Liquid	*B. licheniformis, B. pumilus, B. subtilis, P. polymyxa*
Piranha	Advanced Nutrients	Liquid	*Bacillus mucilaginous, Pseudomonas chlororaphis, P. putida, Trichoderma harzianum, Trichoderma viride*

Another strategy to improve the reliability of microbial inoculants is to start at the source, carefully considering how we capture soil isolates and prospect for traits of interest—from plant growth promotion to niche breadth. One such consideration is the origin of the microbial source pool. Native inoculants have been shown to achieve better success in their native soil contexts than commercial inoculants [126–129] and are expected to be pre-adapted to local climates and soil conditions. The same pattern has also been observed for mycorrhizal fungi, where inocula sourced from local reference ecosystems colonize roots better than commercial inoculants [130]. This is in line with endemism observed in soil communities across geographic ranges [131, 132]—despite the fact that many soil microbes appear to be multidimensional generalists [133]. Given that local isolates often perform better in local contexts than non-native strains, inoculant development efforts that leverage local isolates may help overcome environmental compatibility issues by virtue of pre-adaption to these conditions. While it may not be feasible to create tailored inoculant products for every possible field condition, local isolates could be implemented at regional or national levels for common soil types, crops, or climatic zones. Furthermore, current inoculant sourcing efforts are often narrow in focus, looking at a handful of isolates from a single focal crop, often from a single region, if not a singular focal site. Scaling up isolation efforts to sample broadly across crops, soils, and regions of interest would not only diversify the potential microbial source pool but would also allow growers to use locally adapted isolates for local solutions.

Local isolates may also be less disruptive to the resident microbiome than a “weedy”, fast-growing commercial generalist. “Microbial weeds”, much like their plant counterparts, can be ecologically aggressive due to their generalist nature and rapid growth; accordingly, they may rapidly dominate the communities they invade [134]. More generally, we must evaluate whether selection for elite inoculants also selects for inoculants with weedy traits that are more likely to be invasive and disrupt their recipient soil communities. Ladau *et al.* [135] present a call to action that we must consider the risk that microbial inoculation and environmental manipulation may create microbial invasions. The ecological integrity of agricultural soils likely depends on finding a “Goldilocks zone” where inoculants can successfully establish and provide benefits, but do not aggressively dominate, spread, or persist in the soil long-term [135]. Identifying taxa that are prone to becoming invasive, and which traits promote passage through key invasion stages like establishment and spread, will further help us predict (and reduce) invasion risk.

Another avenue for improving inoculant sourcing efforts is through more intentional and diversified capture strategies. By intentionally capturing generalists and specialists, for instance, we can exploit their unique and complementary strengths. Generalists may exhibit niche breadth in resource use, habitat occupation, host association, cooperative range, dispersal strategies, and their functional roles within communities [136]. This wide niche breadth could make many generalists well-suited to tolerate the production process and diverse recipient environments. However, although generalists may tolerate a broader range of conditions, generalists are expected to be outcompeted by narrowly adapted specialists in a specialist’s “home” environment [136]. Conversely, specialists offer the distinct advantage of high performance in their preferred environments; but may only be effective over a narrow environmental range. Furthermore, focusing solely on isolates already well-adapted to specific environments may narrow the source pool to certain genera, which simultaneously limits the gene pool and target traits available for exploitation [137]. We could, however, mitigate the inherent disadvantages associated with both generalist and specialist lifestyles during development. For instance, the narrow range of individual specialists could be overcome by combining them into consortia to create products that retain suitability for a wide environmental range. Moreover, both generalist and specialist candidate inoculants could be adapted toward improved performance in target environments through ALE during the development process.

Multiple approaches can be used to deliberately capture generalists and specialists of interest. Environmental interfaces, such as riverbeds where soil microbiomes interact with river water, have recently been simulated in microcosms to capture habitat-switching generalists [138]. Regardless of whether these environmental interfaces are directly relevant to the production landscapes that inoculants will face, sampling at these interfaces may enrich for habitat type generalists that could have the ability to persist across other new habitats beyond their isolation source. Conversely, introducing soil communities into the rhizosphere of target crops during microbial capture represents a more trait-specific capture approach, allowing us to preferentially isolate microbes adapted to a specific plant host environment [88, 139]. Microbiome selection could also be used to capture isolates with desired host associations, lifestyles, and traits. Plant-guided soil microbiome selection has been demonstrated with the model plant *Arabidopsis*, where researchers selected and passaged soil microbiomes that enhanced desired plant traits—in this case, early or late flowering times [140, 141]. The late-flowering-associated microbiomes showed enhanced nitrogen mineralization activity and resulted in increased plant biomass [140]. Similarly, wild tomato plants were able to selectively engineer insect-suppressive rhizosphere microbiomes that (temporarily) reduced aphid population size and feeding damage [142]. Plant-guided microbiome selection has also been used to improve leaf greenness [143] and salt tolerance [144] in *Brachypodium distachyon*, and drought tolerance in wheat [145]. Microbiome selection can also be performed in the absence of a host, selecting for traits like amylolytic activity [146], pollutant degradation [147], or low CO_2_ emissions [148]. An obvious extension of this technique would be to isolate candidate inoculants following plant- or trait-guided microbiome selection to select phenotypes of interest. For instance, passaging microbiomes through fluctuating temperatures would select only for those microbes that can thrive under all scenarios, which may enrich for isolates that are robust for application under many temperatures and resilient to in-field temperature oscillations. In contrast, repeatedly exposing communities to a single environmental condition during isolation may select for specialists, which could subsequently be isolated and screened for target traits.

The nutrients used in microbial capture will also constrain which microbial types are selected, as different limiting resources can strongly influence family-level composition across microbiomes obtained from diverse habitats [149]. Intentionally diversifying the media and nutrients used in microbe capture may further allow us to deliberately select microbes with diverse substrate preferences. Microbes face trade-offs that limit their ability to optimize their growth on both glycolytic (e.g. sugars) and gluconeogenic (e.g. organic acids) substrates. As a result, substrate generalists typically demonstrate suboptimal growth under both conditions (compared to specialists) [150]. Substrate supply can thus determine both the structure of microbial communities, and the ratio of substrate generalists and specialists [151]. Microbiomes cultured from soil and supplied with gluconeogenic substrates can be species-poor and dominated by generalists, or species-rich and enriched with specialists, depending on the substrate supplied [151]. In contrast, glycolytic substrates can sustain both specialists and generalists in the same community [151]. In microbiomes where generalists and specialists co-exist, generalists appeared to consume common metabolic byproducts produced by specialists [151]. By deliberately supplying communities with either glycolytic or gluconeogenic substrates, we may therefore be able to select for communities with differing proportions of substrate generalists and specialists.

Currently, inoculant development takes a “bottom-up” approach, where individual strains are isolated and then assembled into consortia. Top–down approaches should also be considered, where communities are cultured and enriched for traits of interest prior to isolation of individual strains [152–154]. Deliberate capture of both individual isolates and communities, using a diverse panel of substrates, could be yet another pathway to optimize the sourcing of diverse inoculants.

## Where generalism is a must (and where it is not)

While some conditions *must* be tolerated by all potential inoculants, such as industrial production and common environmental stressors experienced in agricultural soils, absolute generalism across all possible soil types and climates is not necessarily required. When naturally occurring generalists cannot be sourced, the strategies we have proposed above to breed specialists or poor-performing isolates toward production landscape generalism could potentially be employed to produce generalists (Fig. 2). However, there are other possible routes toward widening environmental breadth.

**Figure 4:**
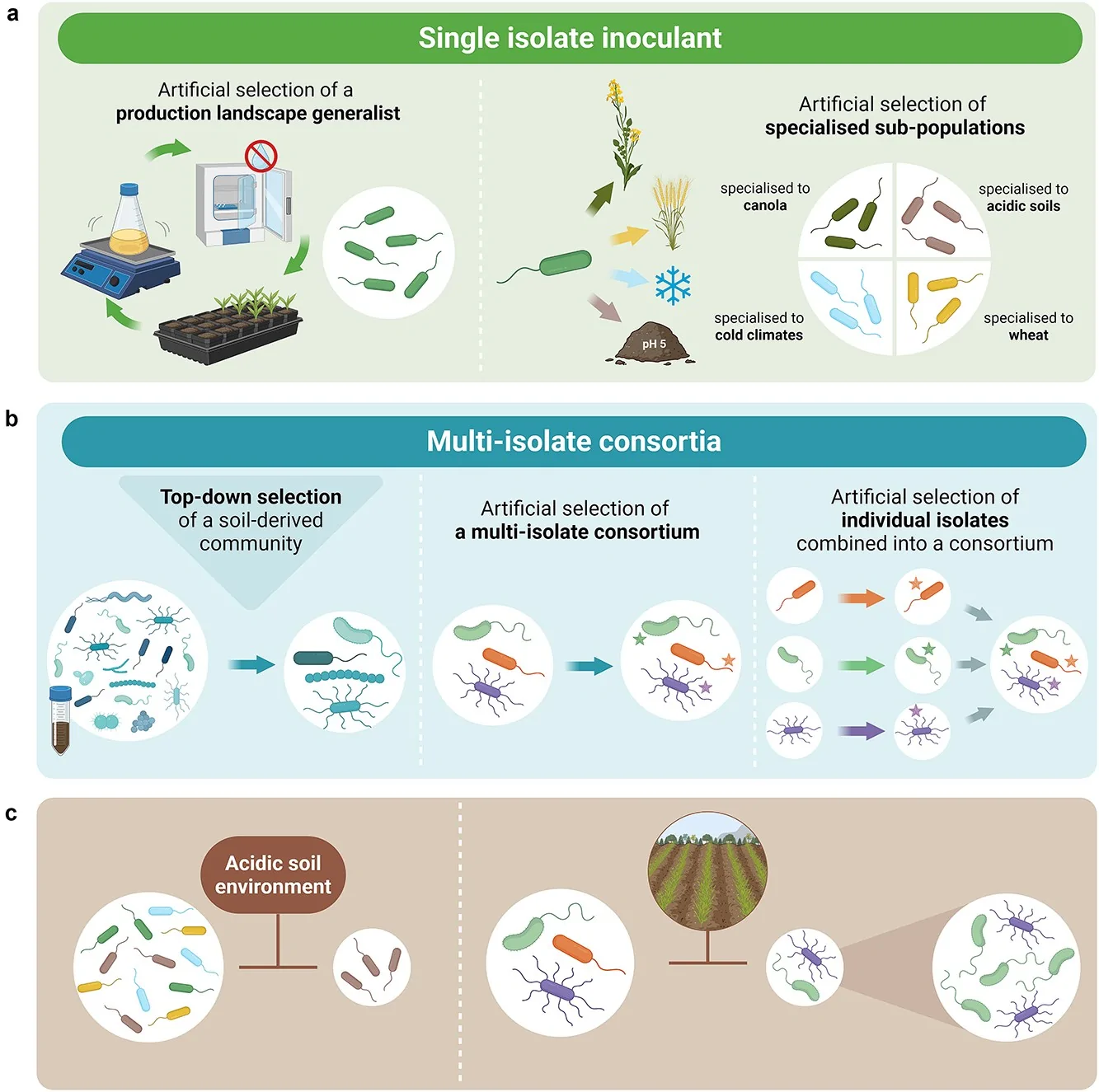
Strategies for developing elite inoculants with a broad environmental range. (a) Developing elite inoculants from single isolates, by selecting for production landscape generalism, or by selecting for specialist sub-populations from a single strain background. (b) Developing elite inoculants from multi-isolate consortia, through top-down selection of soil-derived communities, or forming synthetic consortia either before or after artificial selection. Specialized subpopulations allow the best-adapted variants to be selected for in each environment. Similarly, combining diverse strains into consortia provides redundancy if some isolates are unable to tolerate the recipient environment. (c) Developing redundant consortia, whether comprised of specialized sub-populations or unrelated isolates, allows diverse recipient environments to select out the most compatible inoculant, where a single inoculant might otherwise fail.

Inoculant stacking can be leveraged to combine specialists in consortia with a collectively broad environmental range, thereby balancing the requisite generalism with per-strain environmental specialism. This could be carried out in a single ancestral background, diversifying a single isolate into specialized subpopulations that are fit across diverse field environments (Fig. 4a). By selecting for diversification of traits from a single ancestor and then combining these variants together into a single consortium, a population where fit variants are able to compensate for those incompatible with a recipient environment could be created. This approach may be particularly useful in scenarios where a target isolate is not compatible with and cannot be co-formulated with other taxa. For example, bactericidal proteins such as tailocins can facilitate both “kin” killing of closely related strains and broader interspecific killing of other genera [155–158]. While killing-mediated competition may not be an ideal trait for co-formulation, this trait could offer a competitive advantage supporting the persistence of producing strains in rhizosphere communities [159].

Classical inoculant stacking in multi-isolate consortia allows us to combine diverse gene pools and phenotypes—where strains are compatible. Rather than be constrained by strain compatibility, combining evolved lineages from the same ancestral background may give us the best of both worlds (Fig. 4a)—allowing us to combine elite phenotypes while reducing “non-self” competition during production. Such an approach represents an alternative to more classical inoculant stacking—already used widely in commercial inoculant products—where different strains, species, and genera are combined into a single formula (Fig. 4b). Alternatively, top–down stacking can select for desired functions from a culturable microbial community, entirely forgoing the individual “breeding” and stacking of isolates—resolving issues of compatibility during selection from a wider community (Fig. 4b). Classical inoculant stacking—of multiple strains or species—provides another opportunity to combine diverse traits (with the potential for refinement either before or after stacking; Fig. 4b). As with our earlier suggestion of evaluating microbe–microbe interactions in field environments, we could also evaluate inoculant compatibility with other candidates early in the development process. Inoculants that are antagonistic toward one another in consortia are not ideal targets, even if they have complementary traits or niche breadths. However, *in vitro* antagonism may not translate to in-field antagonism. Recent work on *Bacillus*–*Trichoderma* interactions shows that strains that demonstrate mutual antagonism on agar can still promote plant growth when co-inoculated on tomato [160]. Under *in vitro* conditions, timing matters to the interaction outcome—*T. guizhouense* and *Bacillus velezensis* do not directly inhibit one another when *B. velezensis* is inoculated directly into established fungal cultures [160]. Whether these potentially antagonistic strains actually co-operate and co-exist in the field remains to be seen. Moreover, evaluating *in vitro* antagonism remains directly relevant for co-formulation. Consortia products are commonly co-cultured. Even if antagonistic members are combined only after individual culturing, in-culture competition can still influence per-strain survival to the point of inoculation in the field. Furthermore, if only a fraction of the strains grown in batch culture co-exist, survive, or provide tangible benefits in the field, complex consortia may be less cost effective than single strain products. Alternatively, inoculants that tolerate each other’s presence, or even synergistically boost growth, may have strong potential as consortia products even if they may initially lack requisite traits. In both scenarios, strain compatibility should be evaluated and tracked in more complex soil and field environments, as both diverse soil properties and resident microbiomes may alter their interactions and performance of intended functions.

Although it is not feasible to produce specific formulations for every possible combination of field conditions, combining sub-populations or inoculants specialized to different field environments presents an equivalent solution, allowing any given recipient environment to select the fittest strains (Fig. 4a and c). In fact, this may already be unintentionally occuring in the field with current consortia products (as exemplified in Table 1). What we propose here is that we consider this environmental variability upfront and design for it more explicitly. Longitudinal biosurveillance in recipient environments may also help us better understand which strains persist and provide in-field benefits. Metagenomic biosurveillance is becoming common place to “track and trace” pathogens across landscapes, including in soil and plant systems [161, 162]. The benefits of this approach are numerous. We can understand the success or failure of inoculants to enmesh into a resident microbiome and, in turn, how inoculation may alter the resident microbiome. Furthermore, we can monitor for the loss of necessary or beneficial traits to evaluate efficacy, and assess the risk of inoculants’ potential invasiveness to improve environmental stewardship. All of this evidence would help further establish an efficacy baseline for microbial inoculants.

## Conclusions and future directions

The transition from controlled production settings to complex field conditions presents a significant hurdle for the development of microbial inoculants, which must demonstrate predictable and reliable establishment and function across diverse environments before they can be widely adopted. To overcome this challenge, future research must prioritize development strategies that simultaneously evaluate inoculant performance across the entire production pipeline while explicitly considering potential trade-offs across production landscapes. Integrating production landscape fitness evaluations with genomic inference and selective microbial breeding will help us to understand the drivers of environmental compatibility and identify currently overlooked traits required for commercial viability through the industrial production process. Moreover, the strategies outlined here hold promise not only for agricultural inoculants but also for developing robust and reliable microbial solutions in other ecosystems and industrial production contexts—from probiotics in human health, to the bioremediation of pollutants in the natural environment—where microbes must persist across diverse landscapes in the face of varied selection pressures.

## Data Availability

The dataset analyzed is available on the Canadian Food Inspection Agency’s Registered Fertilizer Products List (https://apps.inspection.canada.ca/webapps/fertilizer).
